# Excision of large right atrial myxoma through a right mini thoracotomy

**DOI:** 10.1093/jscr/rjac182

**Published:** 2022-05-17

**Authors:** Soichiro Henmi, Andrea Salica, Raffaele Scaffa, Salvatore D’Aleo, Lorenzo Guerrieri Wolf, Ruggero De Paulis

**Affiliations:** Cardiac Surgery Department, European Hospital, Rome, Italy; Division of Cardiovascular Surgery, Japanese Red Cross Kobe Hospital & Hyogo Emergency Medical Center, Kobe, Japan; Cardiac Surgery Department, European Hospital, Rome, Italy; Unicamillus University, Roma, Italy; Cardiac Surgery Department, European Hospital, Rome, Italy; Unicamillus University, Roma, Italy; Cardiac Surgery Department, European Hospital, Rome, Italy; Unicamillus University, Roma, Italy; Cardiac Surgery Department, European Hospital, Rome, Italy; Unicamillus University, Roma, Italy; Cardiac Surgery Department, European Hospital, Rome, Italy; Unicamillus University, Roma, Italy

## Abstract

Cardiac myxoma is the most common primary benign cardiac tumor in adults and right atrial myxoma is a rare observation. We report a case of a 56-year-old woman who presented with dyspnea and diagnosed with a right atrial myxoma. Urgent operation through a right mini thoracotomy was done and myxoma was completely excised. Traditionally, median sternotomy with cardiopulmonary bypass is used for excision of cardiac myxoma. Excision through a mini thoracotomy for patients with right atrial myxoma appear to be safe, feasible and efficacious.

## INTRODUCTION

Cardiac myxoma is the most common primary benign cardiac tumor in adults and most of them are located in left atrium [1]. Right atrial myxoma is a rare observation, with an incidence of 0.0017% in the general population [2]. Even though it is benign, some clinical presentations, such as intracardiac obstruction, thromboembolism, cardiac failure or general symptoms such as fatigue, fever and weight loss, could be present.

Traditionally, excision of cardiac myxoma has been executed by median sternotomy with cardiopulmonary bypass (CPB). The approach of right mini thoracotomy has been applied in valve surgery during the last decade. In the present article, we report the successful case of excision of right atrial myxoma through a right mini thoracotomy.

## CASE REPORT

**Figure 1:**
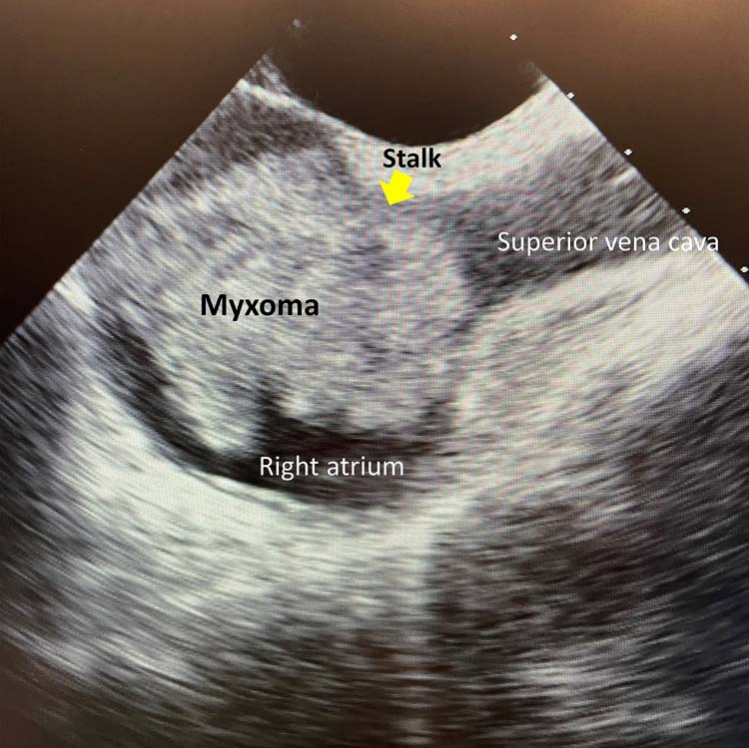
Preoperative transesophageal cardiac echocardiography.

A 56-year-old woman without any past medical history complaining of dyspnea was consulted at our hospital. A contrast-enhanced computed tomography revealed a non-enhanced large mass in the right atrium and pulmonary embolism in the distal part of the left inferior pulmonary artery. Preoperative transesophageal echocardiography (TEE) showed the large highly mobile mass measuring 40 × 41-mm length in the right atrium. The mass attached to the interatrial septum near the ostium of superior vena cava and the diameter of the stalk was 8 mm (Fig. 1). The patient was hemodynamically stable and her saturation of pulse oximetry was 98% at room air.

**Figure 2:**
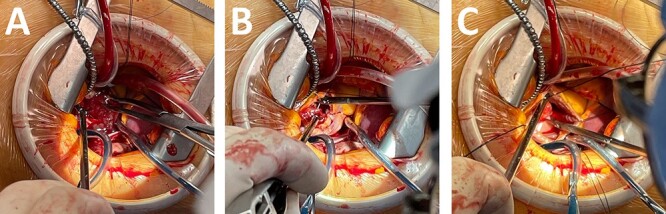
Intraoperative picture. (**A**) Large right atrial myxoma. (**B**) Traction suture at myxoma. (**C**) Resection the stalk of myxoma.

Urgent operation through a right-side mini thoracotomy was performed. After induction of general anesthesia, she was intubated. A venous drainage catheter was placed percutaneously into the right jugular vein before skin incision. She was positioned with the right side of the chest ~30° and with the right arm tucked at the side. The fourth intercostal space was entered with 6-cm skin incision, and a soft tissue retractor was used. Arterial access was achieved with right femoral artery cannulation. Additional venous drainage was achieved with a femoral multistage cannula advanced into the inferior vena cava under TEE guidance, taking care not to enter the right atrium. After CPB was started, aortic cross clamping was achieved by means of a flexible clamp inserted directly through the incision. After cardiac arrest with antegrade blood cardioplegia, the right atrium was incised parallel to the interatrial groove. A gelatinous floating mass appeared (Fig. 2A). The stalk was located in the inter-arterial septum. Traction suture was placed near the stalk of mass (Fig. 2B) and four stay sutures were placed on the right atriotomy to achieve a good exposure. The pedicle of the mass was excised completely (Fig. 2C), and the wall of right atrium was reinforced by surgical stiches. Aortic cross clamp time was 30 min and CPB time was 55 min. Blood transfusions were not necessary. Postoperative course was uneventful. The histopathology revealed the mass to be a myxoma.

## DISCUSSION

Myxoma is the most prevalent primary cardiac tumor and most of them arise in the left atrium. The right atrial myxoma is an unusual location and is the site of 4–11% of cases of cardiac myxoma [1, 3, 4].

The surgical principles of myxoma excision should include exposure of the stalk of the tumor, allowing excision of adequate tissue margins, removal of the tumor, reconstruction of atrial wall defects and the ability to inspect the cardiac chambers for other tumors. Therefore, traditionally excision of cardiac myxoma has been operated by median sternotomy with CPB. In our case, preoperative TEE clearly revealed exact location of the stalk, including the size of the mass. This finding supported us to operate using a minimally invasive approach. Good exposure was easily achieved with four stay sutures placed on the right atriotomy without the need for arterial retractor (Fig. 2). Operation through mini thoracotomy for patients with right atrial myxoma could be easily carried out by every surgeon using this simple setting. In fact, give the favorable position of the right atrium, video assistance was not necessary, nor an atrial retractor.

Regardless of the surgical approach, the ideal resection encompasses the tumor and a portion of the cardiac wall or atrial septum to which it is attached. Whether excision of full-thickness wall is necessary or excision of only an endocardial attachment is sufficient to prevent recurrence is controversial. Several surgeons reported only partial thickness resection of the cardiac wall involved by the tumor claiming that it did not increase the recurrence rate [5, 6]. Our policy was to resect full-thickness wall whenever possible. Therefore, in this case we resected the tumor and the stalk including a part of the interatrial septal thickness.

A higher risk of embolization has been reported and embolic events occurred from 24 to 32% of the patients with cardiac myxoma [3, 4]. Despite it is a rare finding for a myxoma to be originated from the right atrium, a few cases of right atrial myxoma complicated with massive pulmonary embolism have been published [7–9]. Abdelaziz *et al.* reported the sudden onset on massive pulmonary embolism due to right atrial myxoma during preoperative TEE [9]. Urgent operation is needed in these kinds of patients because of the potential risk of massive pulmonary embolization due to the presence of a mobile large mass.

## CONFLICT OF INTEREST STATEMENT

None declared.

## FUNDING

None.
